# Combined nandrolone and resistance training induced cardiac remodelling and oxidative stress despite enhanced cardiomyocyte contractility

**DOI:** 10.1371/journal.pone.0340574

**Published:** 2026-01-08

**Authors:** Alexa Alves de Moraes, Pedro Zavagli Suarez, Arthur Eduardo de Carvalho Quintão, Beatriz Lana Fontes, Sebastião Felipe Ferreira Costa, Carolina Camargos Rocha, Leôncio Lopes Soares, Luciano Bernardes Leite, Leandro Licursi de Oliveira, Emily Correna Carlo Reis, Edilamar Menezes de Oliveira, Pedro Forte, Antônio José Natali, Miguel Araujo Carneiro-Júnior

**Affiliations:** 1 Laboratory of Exercise Biology, Department of Physical Education, Federal University of Viçosa, Viçosa, Brazil; 2 Department of Veterinary Medicine, Federal University of Viçosa, Viçosa, Brazil; 3 Laboratory of Structural Biology, Department of General Biology, Federal University of Viçosa, Viçosa, Brazil; 4 Laboratory of Biochemistry and Molecular Biology of Exercise, School of Physical Education and Sport, University of São Paulo, São Paulo, Brazil; 5 Research Center for Physical Activity and Wellbeing (Livewell), Instituto Politécnico de Bragança, Bragança, Portugal; 6 Department of Sports, Higher Instituto of Educational Sciences of the Douro, Penafiel, Portugal; 7 Department of Sports Sciences, Instituto Politécnico de Bragança, Bragança, Portugal; Emory University School of Medicine, UNITED STATES OF AMERICA

## Abstract

**Background:**

Nandrolone decanoate (ND) is widely used by individuals engaged in resistance training (RT), yet their combined effects on cardiac function remain unclear.

**Objective:**

To investigate the effects of RT and ND on cardiac structure and function, cellular contractility, Ca² ⁺ -handling protein expression, and redox balance in rats.

**Methods:**

Thirty-two male Wistar rats were assigned to four groups: control (C), trained (C-T), ND (N), and trained ND (N-T). Animals received ND or saline for eight weeks, and RT was performed 3 × /week. Cardiac function was assessed by echocardiography, and isolated cardiomyocytes from the left and right ventricles (LV and RV) were evaluated for contractile function. Protein expression of Ca² ⁺ -handling regulators and oxidative stress markers was quantified.

**Results:**

N-T increased LV and RV diameters by 25% and 33%, septal thickness by 41.7%, and reduced ejection fraction by 12.3% compared to N (p < 0.05). LV cardiomyocytes increased width by 29% and volume by 23% in N-T versus C-T (p ≤ 0.04). In LV, N-T showed greater contraction amplitude and velocity at 5 Hz (p = 0.04) and increased relaxation velocity at 1, 3, and 5 Hz (p < 0.01), with shorter time to peak contraction and 50% relaxation at 1 Hz versus N (p = 0.04). In RV, N-T increased contraction amplitude and velocity at 1 and 5 Hz, reduced time to peak at 1 and 3 Hz, shortened relaxation time at 1 Hz, and showed higher relaxation velocity than N and C-T (p < 0.02). Molecular analyses revealed reduced RyR2 expression (25% in LV and 9% in RV) and a 41% decrease in LV phospholamban in N-T versus C-T (p < 0.05). N-T also exhibited higher LV malondialdehyde compared to C-T (p = 0.03).

**Conclusion:**

ND combined with RT induced adverse cardiac remodeling and impaired ventricular function, despite enhanced cardiomyocyte contractility, and intensified molecular and oxidative disturbances, indicating a maladaptive cardiac response. This is the first study to demonstrate regional differences in contractility and redox balance of isolated ventricular cardiomyocytes under ND plus RT.

## Introduction

The widespread use of anabolic-androgenic steroids (AAS) has been increasingly observed among athletes and individuals engaged in physical activity, aiming to enhance strength and muscle mass, thereby improving both athletic performance and physical appearance [1].

The only global meta-analysis available on the epidemiology of AAS use estimated that approximately 3.3% of the world’s population has used such substances, including 6.4% of men and 1.6% of women [2]. However, these figures likely underestimate the real prevalence due to factors as the illicit and stigmatized nature of AAS use, underreporting bias in self-reported surveys, and the prohibition of medical prescriptions for non-clinical purposes in many countries, which favours the clandestine use [3]. More recent regional studies, however, have reported higher prevalence rates —ranging from 3–10% in individuals attending gyms [4] to over 40% among resistance training (RT) practitioners [5] — suggesting that AAS use may have become more widespread in recent years [3].

Such substances derive from testosterone and its analogs, and their metabolic effects include maintaining a positive nitrogen balance, increasing anabolism rate and reducing protein catabolism [6]. Within this context, nandrolone decanoate (ND) stands as one of the most commonly administered AAS [7], likely due to its low cost and relative ease of access compared to other AAS.

Nevertheless, these substances abuse may lead to adverse effects across multiple organ systems [7,8]. In the cardiovascular system specifically, morphophysiological alterations can impair cardiac mechanics and function, including concentric pathological hypertrophy, reduced ventricular compliance, myocardial interstitial fibrosis, and impaired diastolic function [9]. Moreover, recent data evidenced that AAS users have a threefold increased risk of acute myocardial infarction, nearly ninefold increased risk of cardiomyopathy, and more than threefold increased risk of heart failure when compared to an age- and sex-matched general population [3].

Currently, AAS use has become particularly prevalent among individuals who engage in RT. In contrast to the cardiovascular adaptations induced by AAS, RT is associated with the development of concentric physiological cardiac hypertrophy, which is driven by pressure overload [10,11].

Both physiological and pathological cardiac adaptations involve alterations in the excitation–contraction coupling mechanism of cardiomyocytes, which regulates the cardiac contraction-relaxation cycle [12]. Several recent studies have investigated the effects of different physical training modalities, as well as cardiac pathologies, on this cellular mechanism [13–16]. However, there is a notable lack of studies specifically examining the effects of AAS administration on isolated cardiomyocytes’ contractility, indicating a clear need for further investigation [17].

In addition to structural and functional alterations in cardiac tissue, oxidative stress plays a key role in the pathophysiology associated with AAS use. The excessive production of reactive oxygen species (ROS), coupled with a reduction in endogenous antioxidant capacity, can compromise cellular integrity and negatively modulate contractile and intracellular signalling functions in cardiomyocytes [18]. Although evidence suggests that either AAS [19] or RT influence the myocardial redox balance, studies investigating the interaction between these two variables remain scarce [20].

Moreover, a significant limitation in the current state-of-the-art is that most studies either focus exclusively on the left ventricle (LV) or do not distinguish between the LV and the right ventricle (RV) in their analyses. As a result, potential regional differences between the ventricles remain underexplored [20–23]. While LV hypertrophy following AAS exposure is well documented, RV adaptations and their hemodynamic consequences remain poorly characterized. Chronic exposure to ND may increase RV afterload and elevate risk factors for pulmonary arterial hypertension, suggesting that the RV could undergo early functional or molecular changes that are not captured by LV-focused analyses [24]. Investigating both ventricles, particularly at the level of isolated cardiomyocytes and redox homeostasis, provides mechanistic insights into chamber-specific adaptations, as each ventricle exhibits distinct anatomical, functional, and metabolic characteristics [25], which may differentially influence their susceptibility to combined AAS and exercise-induced adaptations.

Thus, this study aimed to investigate the effects RT combined with ND on cardiac morphophysiology, mechanical properties, expression of cardiac calcium-handling proteins and redox balance in left and right ventricles of Wistar rats.

## Methods

### Experimental animals and nandrolone decanoate administration

All procedures were conducted in accordance with the Brazilian Guidelines for the Care and Use of Animals in Teaching or Scientific Research Activities (Brazil, 2022), and were approved by the Ethics Committee on Animal Use of Federal University of Viçosa, under protocol number 44/2022.

Twelve-week-old Wistar rats were provided with water and food *ad libitum* and housed in a room maintained at an average temperature of 22ºC, under an inverted 12-hour light/dark cycle, with the dark period starting at 06:00. Animals were randomly assigned into four groups: sedentary control (C), control + RT (C-T), sedentary ND (N) and ND + RT (N-T). A total of 32 rats were used, with 8 animals per group.

The sample size was determined based on the expected differences in cardiomyocyte contraction amplitude, which was the primary variable for this study. The calculation followed the equation described by Armitage and Berry (1987) [26]:


N = 2 [(Z2α+Z2β)σ/δ]0

Where 2α is the significance level, 2β is 1 – the test power, Z₂α and Z₂β are the corresponding quantiles of the normal distribution, σ is the standard deviation, and δ is the minimum difference to be detected. Using a significance level of 5% (α = 0.05) and a power of 90% (β = 0.1), besides adopting the standard deviation (σ = 0.22) and expected difference (δ = 0.05) obtained from a previous study [27], the sample size was estimated as N = 8.14. Therefore, eight animals per group were considered sufficient to detect statistically significant differences with 90% power for the proposed analysis.

The experiment was conducted in two separate cohorts: one cohort for assessment of isolated cardiomyocyte contractility, and the other cohort for the remaining outcomes. Animals were monitored daily by trained personnel, who had received specific training in rodent handling, injections, and welfare assessment, to minimize suffering and distress. Analgesics were not administered, as the procedures used (ND administration and RT protocol) were not expected to induce acute pain under supervised conditions; veterinary care was available if needed.

The experimental timeline comprised a 2-week RT adaptation period followed by 8 weeks of RT and/or ND administration; thus, the total duration of the experiment was 10 weeks. Both N and N-T received 10 mg/kg of ND (Deca-Durabolin®; Organon LTDA, São Paulo, Brazil) twice per week, totaling 20 mg/kg, administered 30 minutes prior to the start of the training session, for 8 weeks. This dosage was chosen to mimic the pattern of drug abuse typically observed in cycles ranging from 6 to 12 weeks [28]. Studies using animal models involving ND administration combined with physical training vary, with weekly doses ranging from 5 mg/kg to 38 mg/kg of ND [17,20,21,23,29]. Thus, 20 mg/kg represents an intermediate dosage within the spectrum of doses applied in previous studies. Animals in groups C and C-T received 0.2 ml/kg of 0.9% sodium chloride solution, twice weekly, 30 minutes before the start of the training session, for 8 weeks. Substances were administered intramuscularly in the posterior thigh region of the animals, alternating sides at each administration.

Predefined humane endpoints were not used, as the study design required all animals to complete the 8-week RT protocol for valid assessment of the planned outcomes. This choice was ethically justified because the selected ND regimen and training protocol are not expected to induce acute, severe suffering under controlled laboratory conditions, and premature removal would compromise data integrity. Animals were monitored daily by trained personnel, and any showing signs of severe distress (e.g., > 20% weight loss, inability to eat or drink, prostration, or respiratory distress) would have been immediately euthanized using the institutionally approved method; however, no animals reached these criteria during the experimental period. All animals were euthanized at the end of the experiment; no animals were found dead.

### Resistance training protocol

The RT protocol was adapted from Hornberger and Farrar (2004) [30] and consisted of climbing a vertical ladder (1.1 m height, 80º inclination), divided into three phases: adaptation, maximum load carrying test (MLCT), and training.

The two-week adaptation phase, performed thrice weekly, involved familiarization with ladder climbing. During the first week, animals climbed voluntarily with 120-second intervals until completing three consecutive climbs. In the second week, rats completed three sets carrying a 15 g apparatus attached proximally to the tail.

Three days post-adaptation, the MLCT was conducted starting at 75% of body weight, incrementally increasing to 90%, 100%, and then by 30 g increments until failure to climb. This test was repeated at the end of week four to adjust training loads and again at the end of the protocol. RT sessions began 48 hours after the initial MLCT and were performed three times per week for eight weeks. Animals carried out four to nine climbs with progressively increasing loads at 50%, 75%, 90%, and 100% of the MLCT plus an additional 30 g, terminating upon failure or completion of nine successful climbs.

### Echocardiography and sample collection

Echocardiography protocols were performed as described by Lavorato et al. (2016) [31], 48 hours after the last training session. Animals were anesthetized with isoflurane (1.5%) in 100% oxygen at a constant flow rate of 1 L/min (Isoflurane, BioChimico, Brazil), and images were acquired with the animals in the lateral recumbent position. Two-dimensional studies with a fast sampling rate of 120 fps in M-mode were conducted using the MyLab™30 ultrasound system (Esaote, Genoa, Italy) with transducers operating at a nominal frequency of 11 MHz. Two-dimensional transthoracic echocardiography and M-mode images were obtained with a scanning speed of 200 mm, adjusted according to heart rate.

Each parameter was measured over three distinct cardiac cycles, and mean value was used for statistical analysis. The thickness of the posterior and anterior walls of the LV and the interventricular septum (at end-diastole and end-systole), the dimensions of LV chamber, as well as the ejection fraction and fractional shortening were measured. Systolic function of RV was assessed by measuring the tricuspid annular plane systolic excursion (TAPSE). For this, M-mode imaging was performed with the cursor positioned at the lateral portion of the tricuspid annulus. This allowed for the measurement of the base-to-apex displacement during systole by identifying the lateral annulus of the tricuspid valve and recording the end-diastolic and end-systolic distances [31].

Forty-eight hours after echocardiographic analyses, animals were euthanized, and their hearts and ventricles were dissected, weighed, and processed for further analyses as detailed below. The right tibia was dissected and its length measured.

### Cardiomyocyte isolation

The heart was connected to a Langendorff retrograde perfusion system, and single LV and RV myocytes were isolated as previously described [32]. Briefly, the heart was perfused via the aorta with Tyrode’s solution containing (in mM; Sigma-Aldrich, USA): 130 NaCl, 1.43 MgCl₂, 5.4 KCl, 0.75 CaCl₂, 5.0 HEPES, 10.0 glucose, 20.0 taurine, and 10.0 creatine, pH 7.4, for approximately 5 minutes. Tyrode’s solution was then replaced by a calcium-free Tyrode’s solution containing 0.1 mM EGTA for 6 minutes. Subsequently, the heart was perfused with Tyrode’s solution containing 1 mg/ml collagenase type II (Worthington, USA) and 0.1 mg/ml protease (Sigma-Aldrich, USA) for approximately 12 minutes. Then, the ventricles were excised from the digested heart and cut into small fragments, which were placed in a conical tube containing the enzymatic solution (collagenase and protease). Cells were mechanically dissociated by gently agitating the tube for 5 minutes. Dispersed cells were separated from undigested tissue by filtration followed by centrifugation. Isolated cells were stored at 5°C until use, within maximum 2–3 hours after isolation. All solutions used during isolation were oxygenated (100% O₂ – White Martins, Brazil) and maintained at 37°C [32].

This isolation method is a well-established and validated protocol that ensures high-purity cardiomyocyte preparations, minimizing contamination by endothelial cells, fibroblasts, or vascular smooth muscle cells. The technique has been extensively used and validated for electrophysiological measurements [14,32–37].

### Cardiomyocytes’ contractile function

Each ventricle’s myocyte contractile function was measured using an edge-detection system (Ionoptix, Milton, USA) mounted on an inverted microscope (Nikon Eclipse TS100, Japan) as previously described [38]. Myocytes were placed in a chamber on the microscope stage and superfused with Tyrode’s solution containing (in mM; Sigma-Aldrich, USA): 137 NaCl, 5.4 KCl, 0.33 NaH₂PO₄, 0.5 MgCl₂, 5 HEPES, 5.6 glucose, 1.8 CaCl₂, pH 7.4 (adjusted with 5N NaOH) at 37°C. Only myocytes exhibiting clear and regular striation patterns (sarcomeres), no spontaneous contraction in the absence of external stimulation, and responding to 1 Hz stimulation with a single contraction were tested [38].

Myocytes were stimulated (Myopacer, Ionoptix, Milton, USA) to contract at progressively increasing frequencies (1, 3, 5, and 7 Hz) using external electrodes, and resulting cell shortening was measured by analyzing video images of the cell using the Ionoptix camera and software (Ionoptix, Milton, MA, USA). Cell shortening was expressed as a percentage of resting cell length.

Myocyte length and width were obtained from video images, and cell volume was calculated as previously described [39].

### Cardiac calcium-handling proteins expression

Ventricles’ samples were carefully excised and immediately weighed on an analytical balance. For each sample, 100 mg of tissue was placed in a pre-chilled microcentrifuge tube and homogenized on ice in 1 mL of phosphate-buffered saline (PBS, pH 7.4) supplemented with a protease inhibitor cocktail (Sigma-Aldrich, St Louis, MO, USA), using an OMNI motorized homogenizer (OMNI International, Kennesaw, GA, USA). Following homogenization, the samples were centrifuged at 10,000 × *g* for 10 min at 4°C. The resulting supernatant was carefully collected and aliquoted and then stored at −80°C until further analysis to prevent protein degradation. The expression of total phospholamban (PLBt), sodium-calcium exchanger (NCX), sarcoplasmic reticulum Ca² ⁺ -ATPase (SERCA2a), and ryanodine receptor type 2 (RyR2) proteins in both ventricles was assessed by sandwich enzyme-linked immunosorbent assay (ELISA), using the following kits: PLB and NCX (ABclonal Technology, Woburn, USA); SERCA2a (AFG Scientific, Northbrook, USA); and RyR2 (Cloud-Clone, Katy, USA). The ELISA procedure was performed according to the manufacturer’s protocols.

Sample concentrations were determined based on a standard curve generated from serial dilutions, in duplicate, of recombinant rat proteins. Absorbance was measured at 450 nm using a microplate spectrophotometer (BioTek Synergy HTX Multi-Mode Reader, Miami, FL, USA). Proteins’ concentrations were calculated based on standard curves generated for each assay and normalized to the initial tissue weight, with results expressed as ng/mg of tissue. All sample processing and analyses were conducted in a blinded manner concerning experimental group allocation to minimize bias.

### Evaluation of redox-related biomarkers

#### Tissue preparation.

For the enzymatic activity assays, each cardiac ventricle stored at −80 ºC (~100 mg) were homogenized (using the Tissue Master 125 homogenizer, OMNI) in 1 mL of phosphate buffer (pH 7.4) and centrifuged for 10 min at 10,000 *g* (12,000 rpm), under refrigeration at 4°C. After that, the supernatants were used to analyse the enzymatic activity of superoxide dismutase (SOD) and catalase (CAT), besides to quantify byproducts of nitrosative and oxidative stress, such as nitric oxide (NO), malondialdehyde (MDA) and protein carbonyl, as well as measuring total antioxidant capacity. The analysis were carried out using an ELISA microplate reader (Multiskan SkyHigh, Thermo Scientific, Waltham, MA, USA) or a spectrophotometer (UV-Mini 1240, Shimadzu).

#### Superoxide dismutase activity.

SOD activity was determined based on the method that measures the reduction of the superoxide anion (O₂⁻) and hydrogen peroxide, resulting in decreased pyrogallol auto-oxidation, as described by Dieterich et al. (2000) [40]. The reaction mixture contained 10 μL of the sample and 170 μL of sodium phosphate buffer (pH 7.8). The reaction was initiated by adding 20 μL of pyrogallol (10 mM) and incubated at 37 ºC for 30 min. Absorbance was measured at 320 nm. SOD activity was expressed in units per milligram of protein, where one unit of SOD is defined as the amount required to inhibit 50% of the pyrogallol auto-oxidation rate [40].

#### Catalase activity.

CAT activity was measured as described by Aebi (1984) [41], by assessing the rate of H₂O₂ decomposition. Briefly, 100 μL of H_2_O_2_ (20 mM) was added to 5 μL of the cardiac sample. After 3 min, 150 μL of ammonium molybdate (32.4 mM) was added to terminate the reaction. Sample blanks were prepared by replacing H_2_O_2_ with sodium phosphate buffer (50 mM, pH 7.4). The values obtained from the test samples were adjusted by subtracting the blank values. A standard curve was generated using serial dilutions of H_2_O_2_ to determine CAT activity. Absorbance was measured at 374 nm using a spectrophotometer. One unit of catalase activity was defined as the amount of enzyme capable of decomposing 1 mmol of H₂O₂ per minute. Results were expressed in units per milligram of protein [41].

#### Nitric oxide production.

NO production was quantified using the Griess reaction. Briefly, 50 μL of the sample were incubated with an equal volume of Griess reagent (1% sulfanilamide, 0.1% N-(1-naphthyl)ethylenediamine, and 2.5% H₃PO₄) at room temperature for 10 minutes [42]. Absorbance was measured at 540 nm using a microplate spectrophotometer (Multiskan GO, Thermo Scientific). NO concentration (μmol/L) was determined based on a sodium nitrite standard curve (0–100 μM), and results were expressed as μmol/L [42].

#### Malondialdehyde determination.

Lipid peroxidation was assessed by measuring MDA, the major end product of lipid peroxidation, as described by Buege & Aust (1978) [43]. Briefly, 0.2 mL of the sample was homogenized in 0.4 mL of a solution containing trichloroacetic acid (15%), thiobarbituric acid (0.375%), and hydrochloric acid (0.6%). The mixture was incubated in a boiling water bath for 40 minutes. After cooling on ice, 0.6 mL of butanol was added, followed by vortexing for 2 minutes and centrifugation at 9000 g for 10 minutes. The precipitate was discarded, and the supernatant was used to measure absorbance at 535 nm using a microplate scanning spectrophotometer (Multiskan GO). MDA concentration was determined based on a standard curve of known concentrations of 1,1,3,3-tetramethoxypropane (TMPO), and results were expressed as μmol/L per mg of protein [43].

#### Protein oxidation.

Protein carbonyl content was determined using the 2,4-dinitrophenylhydrazine (DNPH) method, as described by Levine et al. (1994) [44], which is based on the reaction of carbonyl groups with DNPH. A total of 50 μL of the sample was added to 0.5 mL of DNPH (10 mmol/L) diluted in 7% hydrochloric acid. The mixture was vortexed and incubated at room temperature, protected from light, for 30 minutes with occasional shaking. Then, 0.5 mL of cold 10% trichloroacetic acid (TCA) was added, followed by centrifugation at 5000 g for 10 minutes at 4°C. The supernatant was discarded, and the pellet was washed three times with 1 mL of a 1:1 (v/v) ethyl acetate/ethanol solution. Finally, 1 mL of 6% SDS was added to the tubes, and the pellet was solubilized by vortexing. Absorbance was measured at 370 nm. Results were expressed as nmol/mg of protein, based on a molar extinction coefficient of ɛ₃₇₀ = 22 mmol/L·cm.

#### Total antioxidant capacity.

Total antioxidant capacity was assessed using the ferric reducing antioxidant power (FRAP) assay, as described by Benzie and Strain (1996) [45], using 2,4,6-tri(2-pyridyl)-s-triazine (TPTZ) as the chromogenic substrate. The method is based on the reduction of the ferric-TPTZ (Fe³ ⁺ -TPTZ) complex to the ferrous form (Fe² ⁺ -TPTZ). A volume of 10 μL of each sample was added to 190 μL of FRAP reagent, which consisted of 25 mL of acetate buffer (300 mmol/L, pH 3.6), 2.5 mL of TPTZ (10 mmol/L), and 2.5 mL of FeCl₃·6H₂O (20 mmol/L). Absorbance was read at 593 nm. The reduction of the Fe³ ⁺ -TPTZ complex by antioxidants was calculated based on a standard curve of serial dilutions of FeSO₄·7H₂O starting at 2 mmol/L. Results were expressed as μmol/mL.

### Statistical analysis

Data were analyzed using two-way ANOVA, followed by Tukey’s post hoc test for multiple comparisons. Relative physical performance at the beginning and end of the experiment were compared through paired t-test. Results were expressed as mean ± standard error of the mean (SEM). Statistical analyses were performed in SigmaPlot (v11.0), with significance set at p < 0.05.

Effect size was calculated using IBM SPSS Statistics 20.0. Eta squared (η²) values were used, with the following interpretation criteria: ≥ 0.01 and < 0.06 – small; ≥ 0.06 and < 0.14 – medium; ≥ 0.14 – large [46].

## Results

### Physical performance test

**Fig 1:**
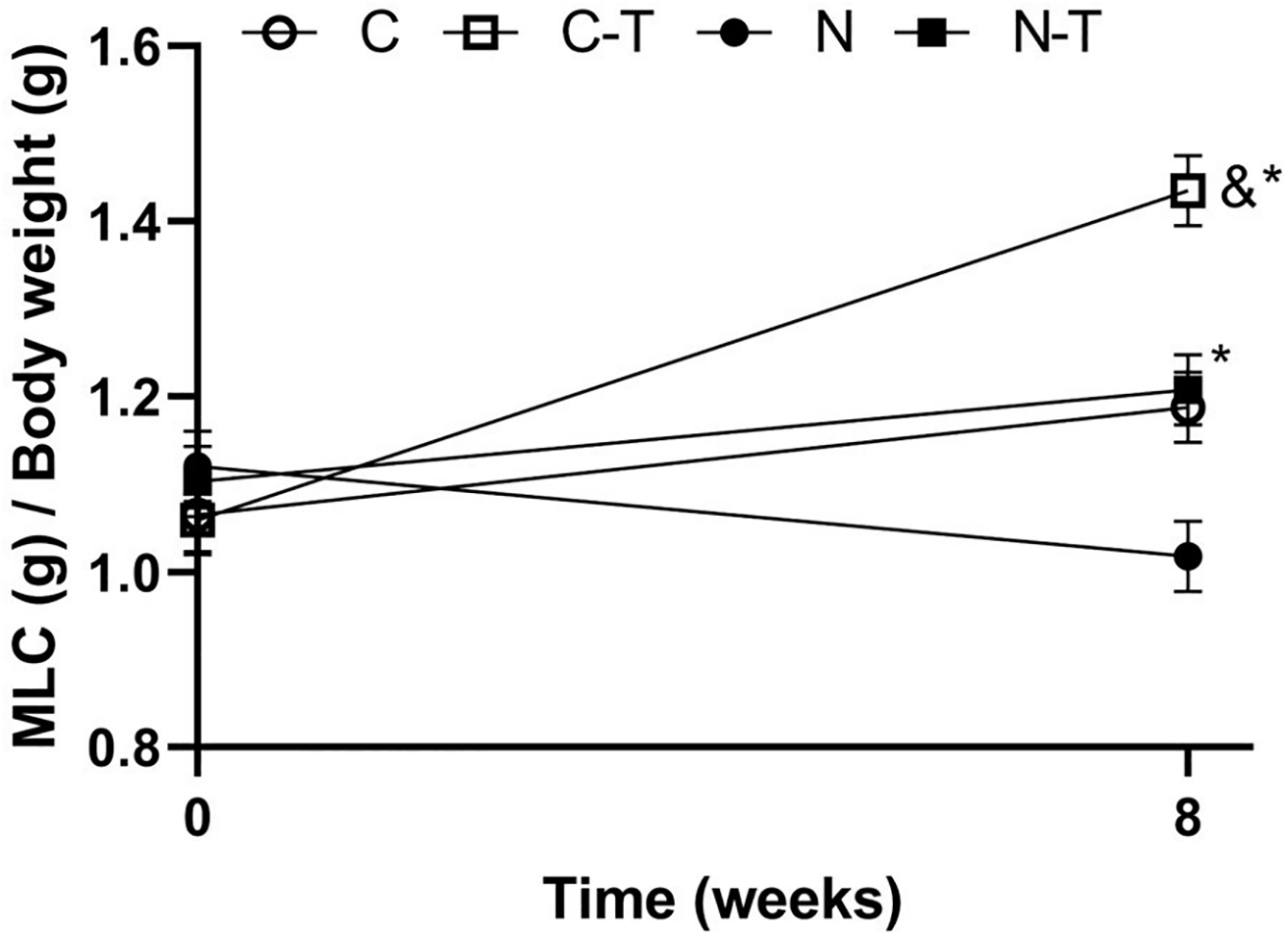
Effects of resistance training and nandrolone decanoate on relative physical performance, expressed as maximum load carried (MLC) divided by body weight, at baseline and after 8 weeks of intervention. Data are presented as mean ± standard error of the mean (n = 8 per group). C, sedentary control; C-T, trained control; N, sedentary nandrolone decanoate; N-T, trained nandrolone decanoate. Paired t-test: & p ≤ 0.05. Two-way ANOVA: * p < 0.05 for resistance training effect.

The results of physical performance tests at baseline and after the experimental period are shown in Fig 1. No differences were observed among groups at baseline. Paired t-test comparisons revealed a significant improvement in physical performance only in the C-T group (p = 0.006). After 8 weeks, no interaction between RT and ND was observed. RT significantly increased physical performance (p = 0.04, η² = 0.16, F = 4.5), whereas ND showed a trend toward performance reduction, with moderate effect size (p = 0.07, η² = 0.13, F = 3.7). No significant intergroups differences were detected.

### Body weight, structural and functional cardiac parameters

Table 1 summarizes data on body weight, and cardiac morphofunctional parameters.

**Table 1:** Effect of resistance training and nandrolone decanoate on body weight and cardiac morphofunctional parameters.

	C (n = 8)	C-T (n = 8)	N (n = 8)	N-T (n = 8)	Interaction RT x ND			Effect of RT			Effect of ND		
					p	η^2^	F	p	η^2^	F	p	η^2^	F
Morphological parameters													
Body weight – baseline (g)	352 ± 16	318 ± 12	319 ± 13	327 ± 14									
Body weight – final (g)	391 ± 16	394 ± 12^&^	360 ± 13^&^	353 ± 14^&.b^	0.06	0.00	0.1	0.87	0.00	0.0	0.02*	0.23^#^	6.7
Heart weight (g)	1.5 ± 0.1	1.9 ± 0.1^a^	2 ± 0.1^a^	2.1 ± 0.1	0.06	0.15^#^	3.8	0.02*	0.21^#^	6.0	0.00*	0.40^#^	14.6
LV weight (g)	0.7 ± 0.06	0.9 ± 0.05^a^	0.9 ± 0.05^a^	0.9 ± 0.06	0.02*	0.22^#^	6.4	0.07	0.14^#^	3.7	0.04*	0.18^#^	5.0
RV weight (g)	0.3 ± 0.03	0.4 ± 0.02^a.d^	0.4 ± 0.03^a.d^	0.5 ± 0.03^b.c^	0.93	0.00	0.0	0.01*	0.30^#^	9.2	0.00*	0.39^#^	14.1
LV weight/Tibial length (mg/mm)	16.4 ± 1.5	22.5 ± 1.2^a^	23.1 ± 1.3^a^	22.3 ± 1.4	0.02*	0.23^#^	6.5	0.06	0.15^#^	3.9	0.02*	0.21^#^	5.9
RV weight/Tibial length (mg/mm)	8.1 ± 0.8	10.3 ± 0.6^a.d^	10.8 ± 0.7^a.d^	13 ± 0.7^b.c^	0.96	0.00	0.0	0.01*	0.28^#^	8.8	0.00*	0.39^#^	13.8
Ecocardiographic data													
RVIDd (mm)	0.8 ± 0.08	0.6 ± 0.07	0.6 ± 0.06	0.8 ± 0.13^c^	0.04*	0.12	4.3	0.4	0.02	0.6	0.8	0.00	0.0
IVSd (mm)	1.5 ± 0.11	1.3 ± 0.15	1.2 ± 0.12	1.7 ± 0.1^c^	0.02*	0.15^#^	5.7	0.2	0.04	1.5	0.8	0.00	0.1
LVIDd (mm)	7.8 ± 0.14	7.5 ± 0.3	7.1 ± 0.4	7.5 ± 0.2	0.28	0.04	1.2	0.8	0.00	0.1	0.3	0.03	1.1
LVPWd (mm)	1.4 ± 0.0	1.6 ± 0.2	1.7 ± 0.2	1.8 ± 0.2	0.9	0.00	0.0	0.4	0.02	0.6	0.2	0.06	1.9
IVSs (mm)	2.9 ± 0.2	2.5 ± 0.2	2.6 ± 0.2	2.6 ± 0.1	0.4	0.02	0.7	0.3	0.03	0.9	0.5	0.02	0.5
LVIDs (mm)	4 ± 0.2	4.3 ± 0.3	3.6 ± 0.3	4.5 ± 0.2^c^	0.2	0.05	1.5	0.02*	0.16^#^	6.1	0.7	0.00	0.2
LVPWs (mm)	2.4 ± 0.2	2.5 ± 0.2	2.5 ± 0.2	2.7 ± 0.1	0.8	0.00	0.1	0.5	0.01	0.4	0.3	0.03	1.1
EF (%)	83.9 ± 2.2	78.8 ± 2.1	85.8 ± 1.9	75.2 ± 2.7^c^	0.2	0.04	1.5	0.00*	0.27^#^	12.2	0.7	0.00	0.1
FS (%)	48.9 ± 2.6	43 ± 2.2	50.7 ± 2.1	39.9 ± 2.3^c^	0.3	0.03	1.1	0.00*	0.29^#^	13.1	0.8	0.00	0.1
TAPSE (mm)	2.5 ± 0.2	2.15 ± 0.1	2.6 ± 0.1	2.1 ± 0.1^c^	0.6	0.01	0.3	0.01*	0.24^#^	7.4	0.9	0.00	0.0

Data presented as mean ± standard error of the mean from 8 animals per group. Groups: C, Untrained control; C-T, Trained control; N, Untrained nandrolone decanoate; N-T, Trained nandrolone decanoate. Paired t-test: & p ≤ 0.05, final body weight – initial body weight. Two-way ANOVA followed by Tukey’s post-hoc test: ^a^ p ≤ 0.05 vs. C; ^b^ p ≤ 0.05 vs. C-T; ^c^ p ≤ 0.05 vs. N; ^d^ p ≤ 0.05 vs. N-T; * p ≤ 0.05; ^#^ large effect size. Abbreviations: EF, ejection fraction; FS, fractional shortening; F, Fisher–Snedecor F distribution; IVSd, interventricular septal thickness in diastole; IVSs, interventricular septal thickness in systole; LVIDd, left ventricular internal diameter in diastole; LVIDs, left ventricular internal diameter in systole; LVPWd, left ventricular posterior wall thickness in diastole; LVPWs, left ventricular posterior wall thickness in systole; ND, nandrolone decanoate; RVIDd, right ventricular internal diameter in diastole; RT, resistance training; RV, right ventricle; LV, left ventricle; TAPSE, tricuspid annular plane systolic excursion; η², eta squared (effect size indicator).

Regarding body weight, C-T, N, and N-T groups exhibited significant increases throughout the experimental period. The C-T group showed the greatest relative gain (+24%), whereas the N-T group had the lowest increase (+8%). At the end of the experiment, no significant differences were found between trained and sedentary groups, while ND significantly reduced final body weight, with a large effect size. In the intergroup comparison, the N-T group had lower body weight than C-T.

Both RT and ND increased heart weight, with a large effect size. The C-T and N groups showed greater heart weight compared to the C group.

For LV weight, a significant interaction between RT and ND was observed, with a large effect size. When analyzed independently, both RT and ND significantly increased LV weight, also with large effect sizes. The C-T and N groups exhibited higher LV weight compared to the C group.

In contrast, no interaction was found between RT and ND for right ventricular (RV) weight. Both factors independently increased RV weight, with large effect sizes. Multiple comparisons showed that the N-T group had greater RV weight than the C-T and N groups, which in turn had higher values than the C group.

The LV hypertrophy index showed a significant RT × ND interaction, with a large effect size. Although RT tended to increase this index, the effect was not statistically significant, despite a large effect size. In contrast, ND led to a significant increase, also with a large effect size. C-T and N groups exhibited higher values than C.

For the RV hypertrophy index, no interaction was found. Both RT and ND increased this index, with large effect sizes. The N-T group had higher values than the C-T and N groups, which were both higher than the C group.

### Ecocardiographic parameters

**Fig 2:**
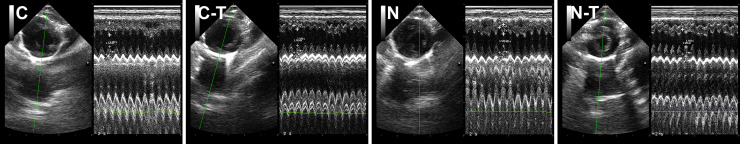
Representative echocardiographic images. C, untrained control; C-T, trained control; N, untrained nandrolone decanoate; N-T, trained nandrolone decanoate.

Echocardiographic analysis (Fig 2 and Table 1) revealed no major morphofunctional alterations induced by ND alone.

A significant interaction between RT and ND was observed for right ventricular internal diameter in diastole (RVIDd) and interventricular septal thickness in diastole (IVSd), with moderate to large effect sizes. However, neither factor exerted isolated effects. Post-hoc analysis showed that N-T exhibited higher RVIDd and IVSd values compared to the N group.

For the left ventricular internal diameter in systole (LVIDs), no interaction was detected, but RT significantly increased this parameter with large effect size. Post-hoc comparison indicated higher LVIDs in the N-T group versus N.

Ejection fraction (EF), fractional shortening (FS), and tricuspid annular plane systolic excursion (TAPSE) were not affected by ND, but were significantly reduced by RT, with large effect size. However, this reduction was evident only in the N-T group when compared to N. No significant main or interaction effects were observed for the other echocardiographic parameters analyzed.

### Cardiomyocyte morphology

**Fig 3:**
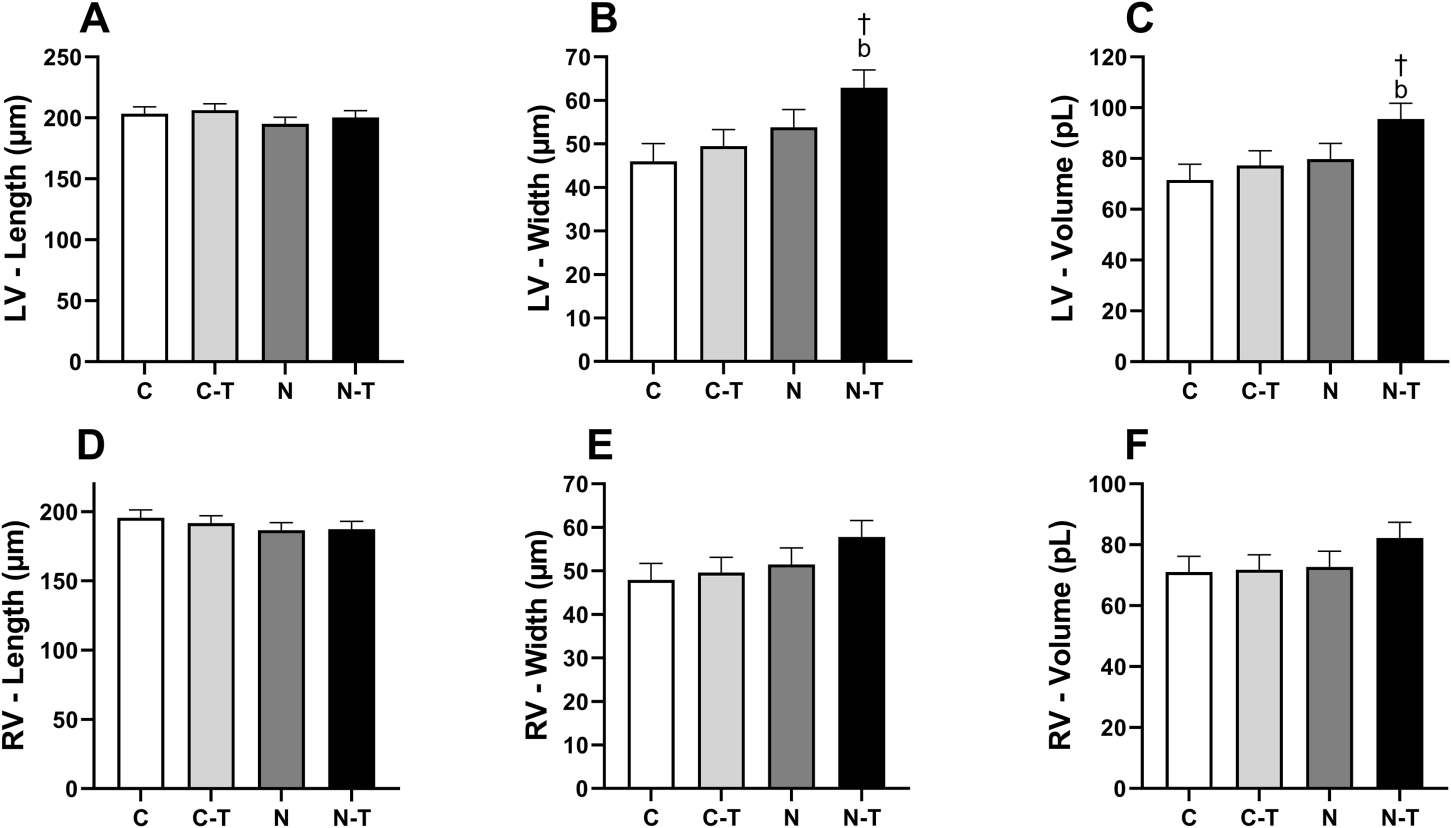
Effects of resistance training and nandrolone decanoate on length, width, and volume of isolated cardiomyocytes from the left and right ventricles. **(A)** Left ventricular cardiomyocyte length (µm); **(B)** left ventricular cardiomyocyte width (µm); **(C)** left ventricular cardiomyocyte volume (pL); **(D)** right ventricular cardiomyocyte length (µm); **(E)** right ventricular cardiomyocyte width (µm); **(F)** right ventricular cardiomyocyte volume (pL). Data are presented as mean ± standard error of the mean from 60 to 80 cells per group. Groups: C, untrained control; C-T, trained control; N, untrained nandrolone decanoate; N-T, trained nandrolone decanoate. b p ≤ 0.05, N-T vs. C-T; † p ≤ 0.05, main effect of nandrolone decanoate. Data analyzed by two-way ANOVA followed by Tukey’s post-hoc test.

Cardiomyocyte length, width, and volume are presented in Fig 3.

In LV cardiomyocytes, no interaction between RT and ND was observed for cell length (Fig 3A), and neither factor alone influenced this parameter. Post-hoc comparisons also revealed no differences between groups.

For cell width (Fig 3B), there was no interaction between RT and ND, and RT alone had no significant effect. However, ND promoted an increase in LV cardiomyocyte width, with a large effect size. This enlargement was confirmed in post-hoc analysis, with the N-T group showing greater width compared to the C-T group. Similarly, cell volume (Fig 3C) showed no interaction between the factors, and RT alone did not affect this variable, although a moderate effect size was noted. ND increased LV cardiomyocyte volume, with a large effect size, and the N-T group exhibited higher values than the C-T group.

In RV cardiomyocytes (Fig 3D-3F), no significant effects or interactions were observed for any of the evaluated morphological parameters, with all analyses indicating small effect sizes. No intergroup differences were detected in post-hoc comparisons.

### Contractile function

**Fig 4:**
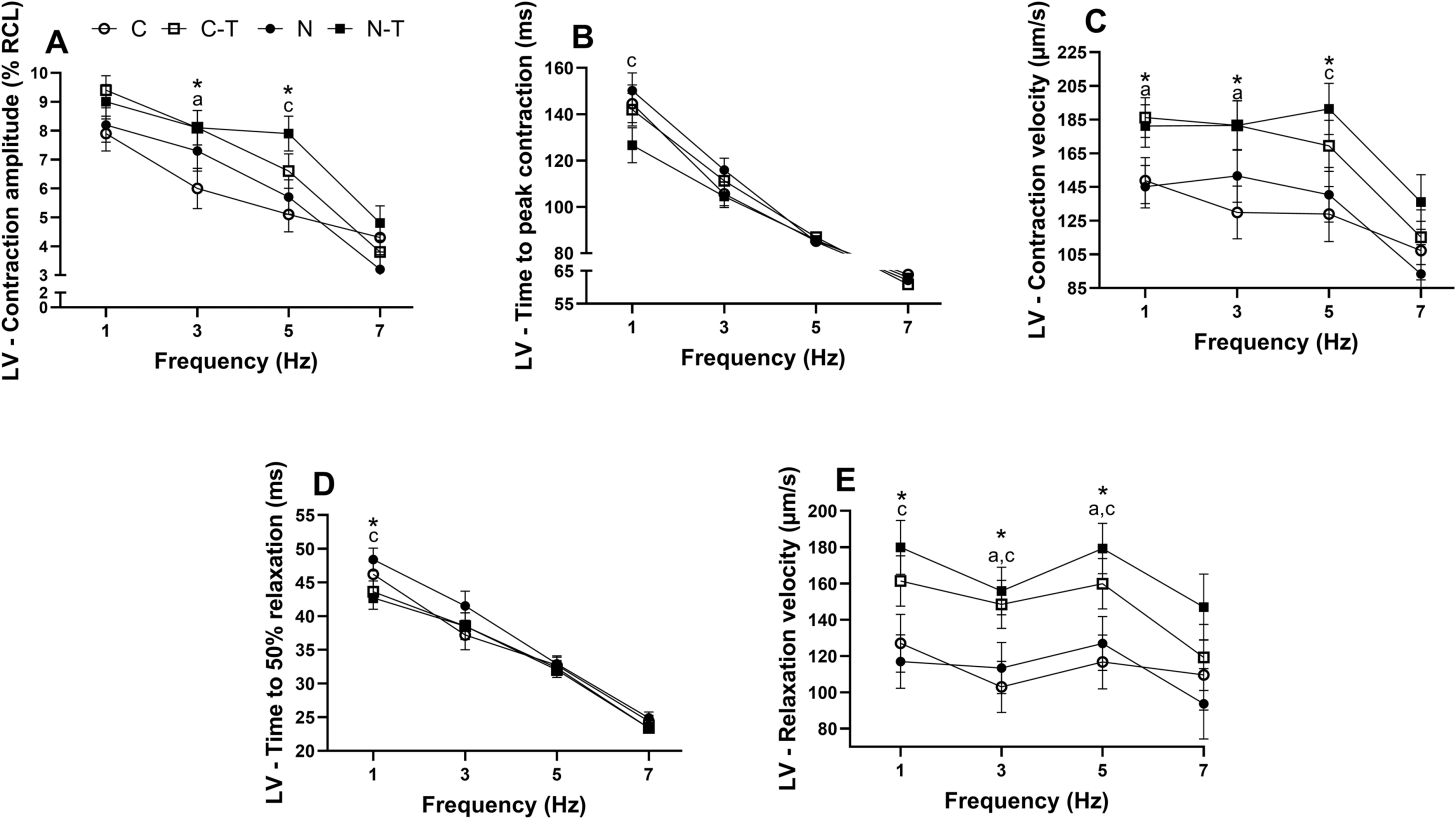
Effects of resistance training and nandrolone decanoate on contractility parameters of left ventricular (LV) cardiomyocytes from Wistar rats electrically stimulated at 1, 3, 5, and 7 Hz. **(A)** Contraction amplitude, expressed as % of resting cell length (% RCL); **(B)** Time to peak contraction (ms); **(C)** Contraction velocity (µm/s); **(D)** Time to 50% relaxation (ms); **(E)** Relaxation velocity (µm/s). Data are presented as mean ± standard error of the mean from 6 to 10 cells per animal (n = 6–8 animals per group). Groups: C, untrained control; C-T, trained control; N, untrained nandrolone decanoate; N-T, trained nandrolone decanoate. a p ≤ 0.05, C vs. C-T; c p ≤ 0.05, N vs. N-T; * p ≤ 0.05, main effect of resistance training; † *p* ≤ 0.05, main effect of nandrolone decanoate. Data were analyzed by two-way ANOVA followed by Tukey’s post-hoc test, except for time to 50% relaxation, which was analyzed using the Kruskal–Wallis test followed by Dunn’s post-hoc test.

Under electrical stimulation (1–7 Hz), LV cardiomyocytes (Fig 4) showed distinct responses depending on the intervention. No interaction between RT and ND was found, and ND alone had no significant effect. RT increased contraction amplitude (Fig 4A) at 3 Hz (p = 0.04) and 5 Hz (p = 0.005), both with large effect sizes. These changes were mainly driven by the C-T group compared to C at 3 Hz, and the N-T group compared to N at 5 Hz. Time to peak contraction (Fig 4B) was unaffected except at 1 Hz, where N-T was shorter than N (p = 0.04). Contraction velocity (Fig 4C) was enhanced by RT at 1, 3, and 5 Hz (all p ≤ 0.01), with large effect sizes. The C-T group showed higher values than C at 1 and 3 Hz, and the N-T group higher than N at 5 Hz. Time to 50% relaxation (Fig 4D) was reduced at 1 Hz in trained animals (p = 0.03), with a large effect size, and N-T was faster than N. Relaxation velocity (Fig 4E) increased with RT at 1, 3, and 5 Hz (all p < 0.01), with large effect sizes. The N-T group showed higher values than N across all frequencies, and the C-T group was greater than C at 3 and 5 Hz.

**Fig 5:**
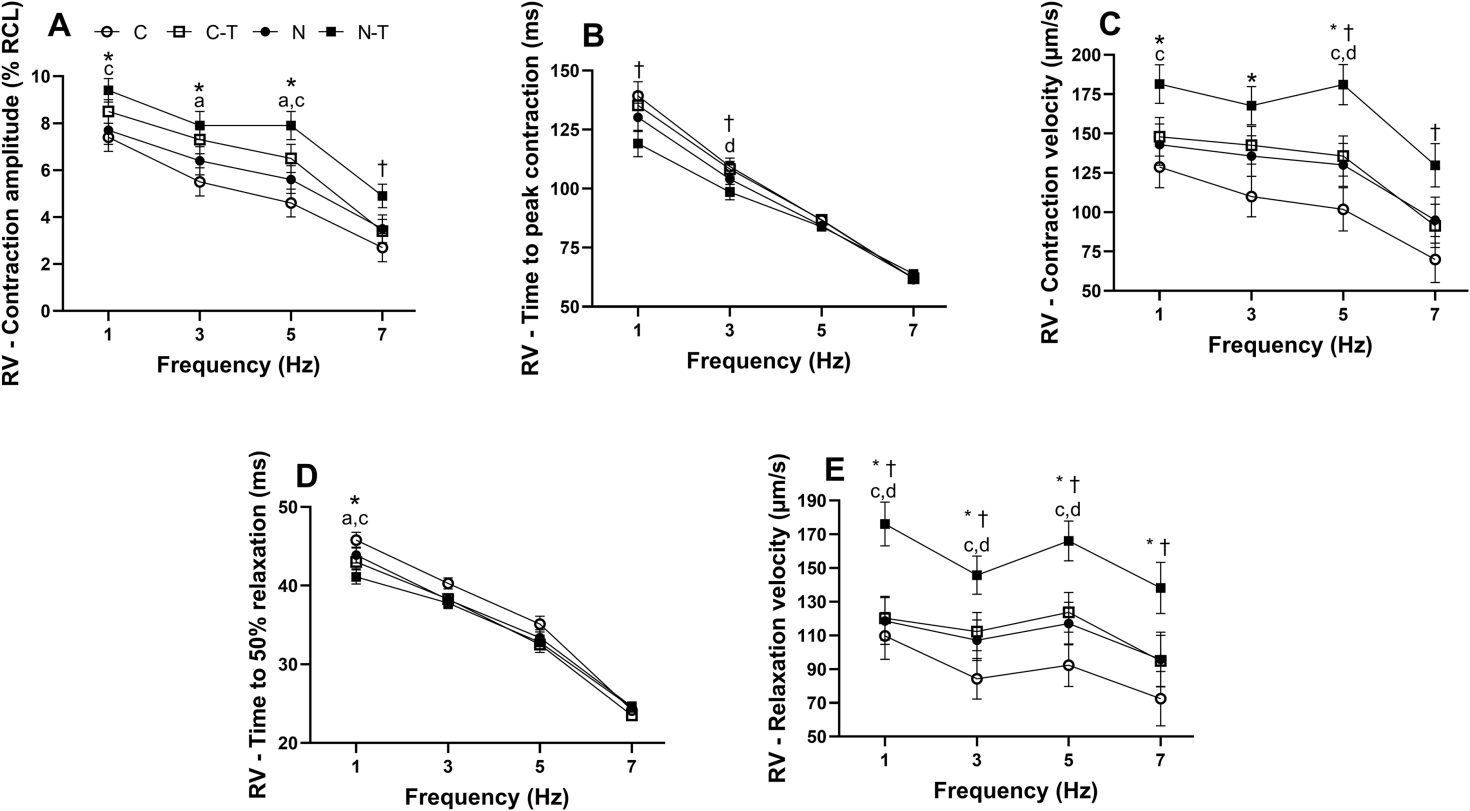
Effects of resistance training and nandrolone decanoate on contractility parameters of right ventricular (RV) cardiomyocytes from Wistar rats electrically stimulated at 1, 3, 5, and 7 Hz. **(A)** Contraction amplitude expressed as % of resting cell length (% RCL); **(B)** Time to peak contraction (ms); **(C)** Contraction velocity (µm/s); **(D)** Time to 50% relaxation (ms); **(E)** Relaxation velocity (µm/s). Data are presented as mean ± standard error of the mean from 6 to 10 cells per animal (n = 6–8 animals per group). Groups: C, untrained control; C-T, trained control; N, untrained nandrolone decanoate; N-T, trained nandrolone decanoate. a p ≤ 0.05, C vs. C-T; c p ≤ 0.05, N vs. N-T; d p ≤ 0.05, C-T vs. N-T; * p ≤ 0.05, main effect of resistance training; † *p* ≤ 0.05, main effect of nandrolone decanoate. Data were analyzed by two-way ANOVA followed by Tukey’s post-hoc test, except for time to 50% relaxation, which was analyzed using the Kruskal–Wallis test followed by Dunn’s post-hoc test.

Regarding RV cardiomyocytes (Fig 5), no significant interaction between RT and ND was observed in any contractility parameter. However, both factors exerted isolated effects. RT increased contraction amplitude (Fig 5A) at 1 (p = 0.02), 3 (p = 0.02), and 5 Hz (p = 0.002), all with large effect sizes. ND also increased this parameter at 7 Hz (p = 0.046), with a moderate effect size. Post-hoc comparisons revealed that the N-T group had higher values than N at 1 and 5 Hz, while the C-T group outperformed C at 3 and 5 Hz. Time to peak contraction (Fig 5B) was unaffected by RT but was reduced by ND at 1 and 3 Hz (both p = 0.04), with large effect sizes. At 3 Hz, N-T was significantly lower than C-T. Contraction velocity (Fig 5C) was increased by RT at 1 (p = 0.03), 3 (p = 0.03), and 5 Hz (p = 0.003), and by ND at 5 (p = 0.01) and 7 Hz (p = 0.03), all with large effect sizes. The N-T group showed higher values than N at 1 and 5 Hz and was also higher than C-T at 5 Hz. Time to 50% relaxation (Fig 5D) was shortened by RT at 1 Hz (p = 0.007), with a large effect size. At this frequency, both N-T and C-T had lower values compared to N and C, respectively. Relaxation velocity (Fig 5E) was increased by both RT and ND at all frequencies analyzed (RT: p < 0.05; ND: p = 0.02), with large effect sizes. Post-hoc analysis showed that N-T had higher values than both N and C-T at 1, 3, and 5 Hz.

### Cardiac Ca^2+^-handling proteins expression

**Fig 6:**
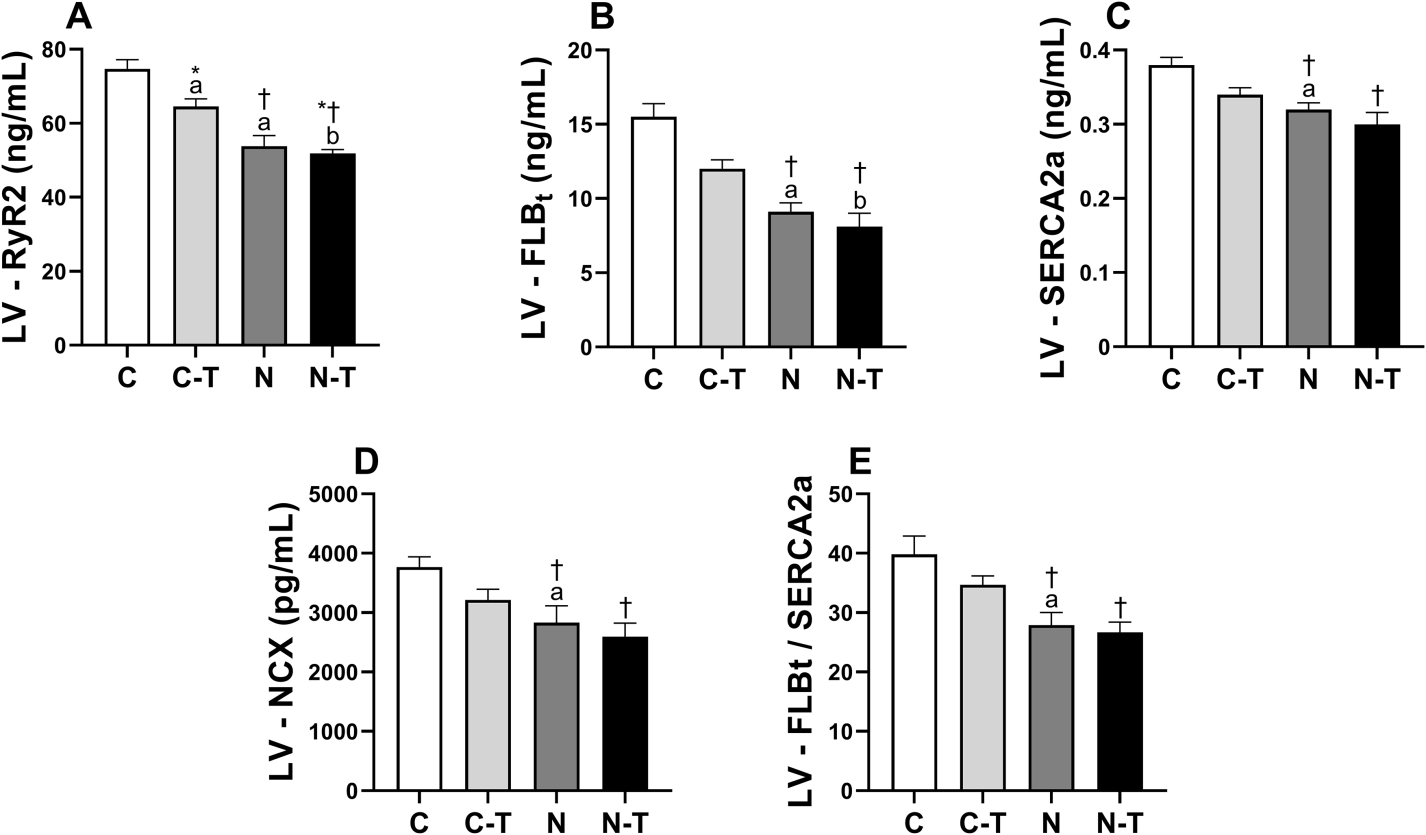
Effects of resistance training and nandrolone decanoate on the expression of calcium-handling proteins in the left ventricle (LV). **(A)** Ryanodine receptor type 2; **(B)** Total phospholamban; **(C)** Sarcoplasmic reticulum Ca² ⁺ -ATPase (cardiac isoform); **(D)** Sodium–calcium exchanger; **(E)** Total phospholamban/ Sarcoplasmic reticulum Ca² ⁺ -ATPase ratio. Data are presented as mean ± standard error of the mean for six animals per group. Groups: C, untrained control; C-T, trained control; N, untrained nandrolone decanoate; N-T, trained nandrolone decanoate. ^a^ p ≤ 0.05 vs. C; ^b^ p ≤ 0.05 vs. C-T; * p ≤ 0.05, main effect of resistance training; † p ≤ 0.05, main effect of nandrolone decanoate. Two-way ANOVA followed by Tukey’s post hoc test.

**Fig 7:**
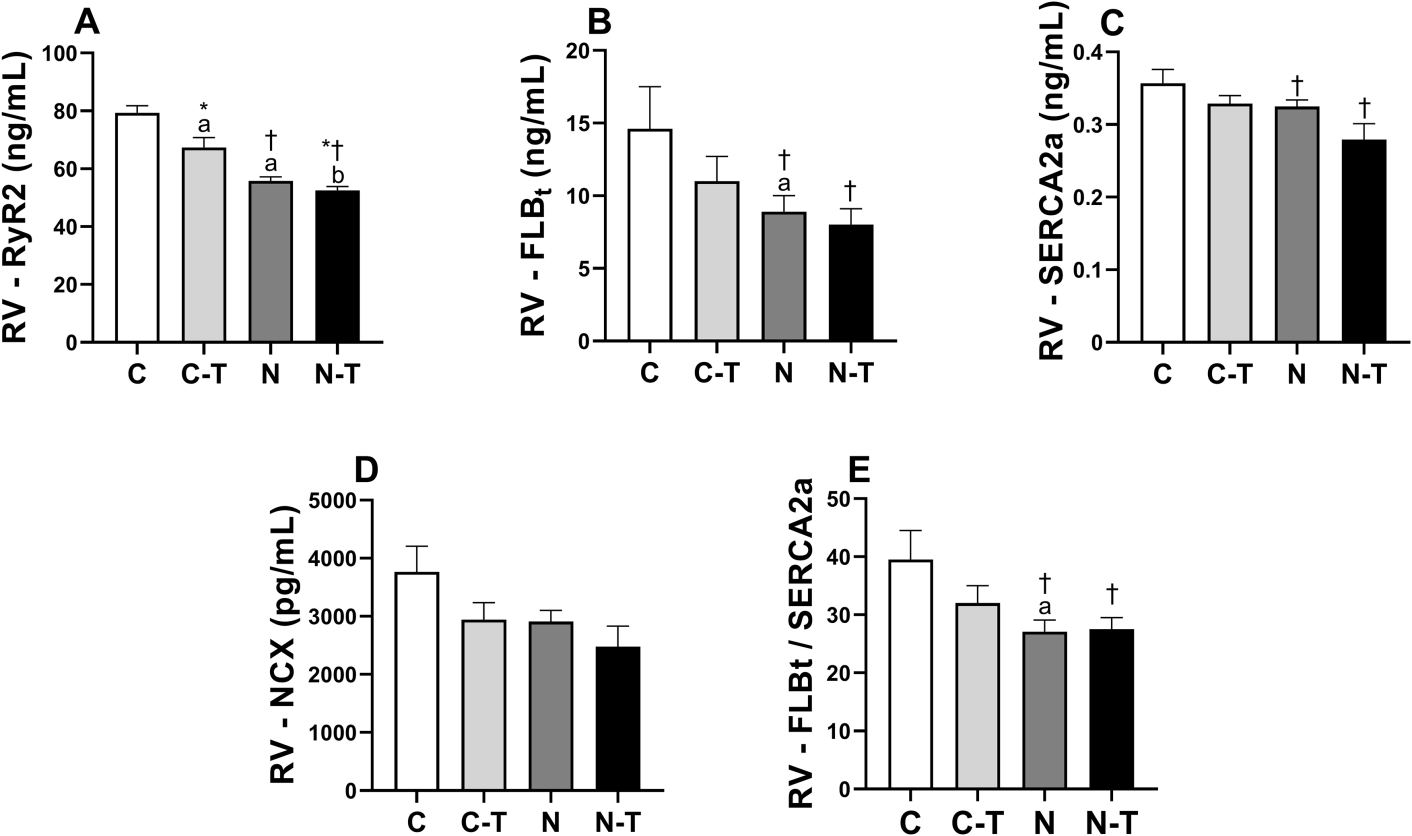
Effects of resistance training and nandrolone decanoate on the expression of calcium-handling proteins in the right ventricle (RV). **(A)** Ryanodine receptor type 2; **(B)** Total phospholamban; **(C)** Sarcoplasmic reticulum Ca² ⁺ -ATPase (cardiac isoform); **(D)** Sodium–calcium exchanger; **(E)** Total phospholamban/ Sarcoplasmic reticulum Ca² ⁺ -ATPase ratio. Data are presented as mean ± standard error of the mean for six animals per group. Groups: C, untrained control; C-T, trained control; N, untrained nandrolone decanoate; N-T, trained nandrolone decanoate. ^a^ p ≤ 0.05 vs. C; ^b^ p ≤ 0.05 vs. C-T; * p ≤ 0.05, main effect of resistance training; † p ≤ 0.05, main effect of nandrolone decanoate. Two-way ANOVA followed by Tukey’s post hoc test.

Cardiac Ca² ⁺ -handling protein expression in LV and RV is shown in Figs 6 and 7. No significant interaction between RT and ND was observed for any of the proteins analyzed.

In the LV, ND consistently reduced the expression of all proteins, with large effect sizes. RyR2 expression (Fig 6A) was reduced by both RT (p = 0.012) and ND (p < 0.001). Post-hoc comparisons revealed that group C had higher values than C-T (p = 0.004) and N (p < 0.001), and C-T was higher than N-T (p < 0.001). ND also reduced the expression of PLBt (Fig 6B, p < 0.001), with C and C-T showing higher values than N (p = 0.002) and N-T (p = 0.04), respectively. SERCA2a expression (Fig 6C) was also reduced by ND (p = 0.006), with C showing higher values than N (p = 0.02). ND decreased NCX expression (Fig 6D, p = 0.02), and C exhibited higher values than N (p = 0.004). Finally, the PLBt/SERCA2a ratio (Fig 6E) was reduced by ND (p < 0.001), with both C and C-T showing higher values than N (p = 0.003) and N-T (p = 0.003), respectively.

In the RV, a similar pattern was observed. RyR2 expression (Fig 7A) was reduced by both RT (p = 0.005) and ND (p < 0.001), with large effect sizes. Group C had higher values than C-T (p = 0.002) and N (p < 0.001), and C-T was higher than N-T (p < 0.001). PLBt (Fig 7B) expression was reduced by ND (p = 0.03), with group C showing higher values than N (p = 0.04). SERCA2a expression (Fig 7C) was also reduced by ND (p = 0.04), with no significant differences in the multiple comparisons. Although RT did not significantly alter SERCA2a, it showed a large effect size (p = 0.07). NCX expression (Fig 7D) showed a non-significant reduction following ND treatment (p = 0.09), though accompanied by a large effect size, with no significant post-hoc differences. The PLBt/SERCA2a ratio (Fig 7E) was reduced by ND (p = 0.049), and group C showed higher values than N (p = 0.042).

### Redox balance

**Fig 8:**
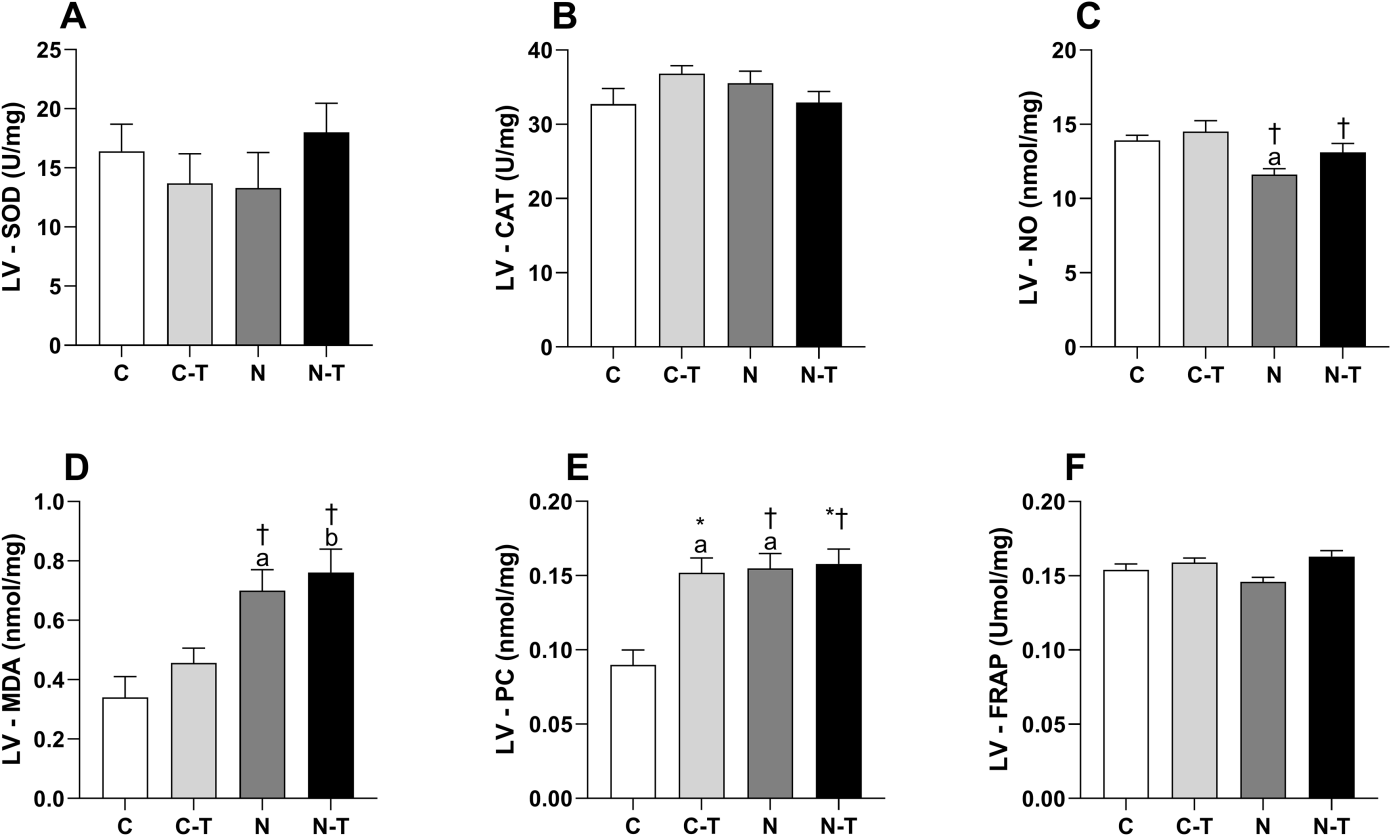
Effects of resistance training and nandrolone decanoate on redox balance markers in the left ventricle (LV). **(A)** Superoxide dismutase; **(B)** Catalase; **(C)** Nitric oxide; **(D)** Malondialdehyde; **(E)** Protein carbonyl content; **(F)** Ferric reducing antioxidant power. Data are presented as mean ± standard error of the mean for six animals per group. Groups: C, untrained control; C-T, trained control; N, untrained nandrolone decanoate; N-T, trained nandrolone decanoate. a p ≤ 0.05 vs. C; b p ≤ 0.05 vs. C-T; * p ≤ 0.05, main effect of resistance training; † p ≤ 0.05, main effect of nandrolone decanoate. Two-way ANOVA followed by Tukey’s post hoc test.

In the LV (Fig 8), no significant interaction was found between RT and ND for any of the variables. SOD (Fig 8A) and CAT (Fig 8B) levels were unaffected by either intervention, with small effect sizes. NO production (Fig 8C) was reduced by ND (p = 0.01) with a large effect size; post hoc analysis revealed lower levels in N vs. C (p = 0.03). MDA expression (Fig 8D) increased with ND (p = 0.001), also with a large effect size, and was higher in both N and N-T compared to their respective controls (p = 0.01 and p = 0.03). PC content (Fig 8E) was influenced by both RT (p = 0.02) and ND (p = 0.01), with a significant interaction between them (p = 0.03), all with large effect sizes. C-T and N groups showed higher values than C (p = 0.002). FRAP (Fig 8F) showed a trend toward increase with RT (p = 0.07), with a large effect size, but no differences were detected between groups.

**Fig 9:**
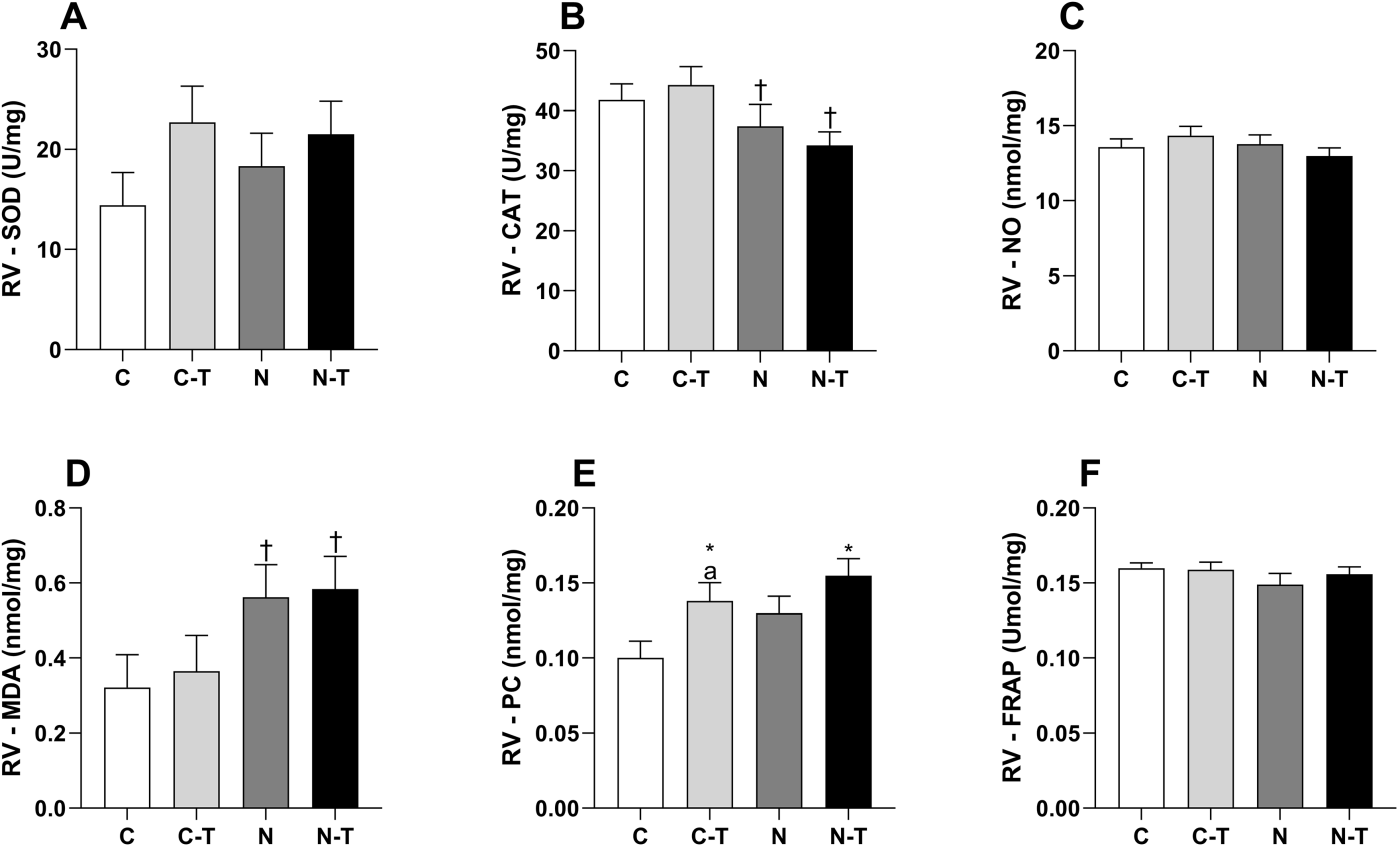
Effects of resistance training and nandrolone decanoate on redox balance markers in the right ventricle (RV). **(A)** Superoxide dismutase; **(B)** Catalase; **(C)** Nitric oxide; **(D)** Malondialdehyde; **(E)** Protein carbonyl content; **(F)** Ferric reducing antioxidant power. Data are presented as mean ± standard error of the mean for six animals per group. Groups: C, untrained control; C-T, trained control; N, untrained nandrolone decanoate; N-T, trained nandrolone decanoate. a p ≤ 0.05 vs. C; * p ≤ 0.05, main effect of resistance training; † p ≤ 0.05, main effect of nandrolone decanoate. Two-way ANOVA followed by Tukey’s post hoc test.

In the RV (Fig 9), SOD (Fig 9A) and CAT (Fig 9B) expression were not significantly changed by either factor, though CAT was reduced by ND (p = 0.01) with a large effect size. NO (Fig 9C) remained unchanged across all groups. MDA (Fig 9D) increased with ND (p = 0.02), with a large effect size, but no significant intergroup differences were detected. PC levels (Fig 9E) increased with RT (p = 0.02), and ND showed a trend toward elevation (p = 0.07), both with large effect sizes; post hoc analysis revealed higher values in C-T vs. C (p = 0.0049). FRAP (Fig 9F) was unaffected by either treatment, with small effect sizes and no intergroup differences.

## Discussion

This study evaluated the effects of RT combined with ND on cardiac morphophysiology, molecular and mechanical properties, and redox balance in isolated cardiomyocytes from both ventricles of Wistar rats. It is a pioneering investigation, as the behavior of isolated cardiomyocytes under the combined influence of anabolic steroids and resistance training remains underexplored.

In Wistar rats, the combination of ND and RT impaired physical performance and limited body weight gain, while inducing hypertrophy in both ventricles and increasing cardiomyocyte’s width and volume in the LV. This combination also led to LV systolic chamber dilation, interventricular septum thickening, and RV diastolic enlargement. Systolic function of both ventricles was reduced, as demonstrated by decreased ejection fraction, fractional shortening, and TAPSE. At the cellular level, ND + RT enhanced contraction and relaxation parameters in both ventricles and reduced the expression of key calcium-handling proteins (RyR2 in both ventricles, PLBt in the LV). Furthermore, N-T group exhibited increased MDA levels in the LV and elevated protein carbonylation in both ventricles. Collectively, these findings suggest that the combined use of ND and RT disrupts redox homeostasis and impairs calcium regulatory mechanisms, contributing to adverse cardiac remodelling and functional decline.

Regarding physical performance, although the interaction between ND and RT was not statistically significant, RT alone significantly improved physical performance in trained rats, consistent with expected neuromuscular adaptations such as increased motor unit recruitment and better motor synchronization, which typically occur early in strength training [47].

In contrast, ND demonstrated a trend toward impairing performance with a moderate effect size, likely related to cardiac dysfunction induced by the steroid. Higher or prolonged ND exposure is associated with greater risk of pathological cardiac hypertrophy, arrhythmias, reduced ejection fraction, and systemic toxicity, which may explain the observed performance decline [48]. Additionally, previous studies reporting performance gains with ND and RT used lower total doses – e.g., 60–80 mg/kg over 4–6 weeks, corresponding to approximately 8 mg/kg/week [20,23] – whereas the present study applied a higher cumulative dose of 160 mg/kg of ND, administered as weekly doses of 20 mg/kg over eight weeks. This dosing was chosen to reflect current patterns of ND abuse, in which many individuals seeking accelerated gains in performance and muscle mass use similar or even higher doses [28,49].

Body weight gain was significantly limited by ND, especially when combined with RT. Although animals in the C-T, N, and N-T groups evidenced significant weight gain over time, factor analysis revealed a strong inhibitory effect of ND on this increase. Among groups, the N-T animals exhibited the lowest final body weight, significantly differing from the N group. Previous studies on AAS have yielded varying results regarding their effects on body weight, with no clear consensus established as there are reports of body weight maintenance [50,51], reduction [52,53], or gain [54]. The observed body mass inhibition here may be related to ND-induced increases in catecholamine expression, which can reduce appetite and thus limit weight gain [55]. Additionally, a possible explanation for this finding is an increase in adipose tissue lipolysis induced by ND [56]. Importantly, while ND alone showed inhibitory tendencies, significant weight differences were found only when combined with RT, suggesting a synergistic effect between the two interventions in limiting body weight gain.

Significant morphological alterations were observed in the hearts of rats subjected to the combined treatment ND and RT. This association notably increased heart weight and the hypertrophy index of the LV, indicating pronounced cardiac remodelling. While both ND and RT independently contributed to both ventricular hypertrophies, their combination potentiated these effects, particularly in the LV.

Cardiac hypertrophy observed in the N-T groups likely results from the convergence of physiological and pathological pathways. RT alone is known to induce physiological hypertrophy through activation of the PI3K/Akt/mTOR pathway, which promotes ribosomal biogenesis and protein synthesis in response to pressure overload, without compromising cardiac function [57].

In contrast, ND triggers maladaptive hypertrophy, mediated by genomic actions through androgen receptor activation and non-genomic effects involving Akt/mTOR and calcium-dependent signalling [58,59]. In turn, excess Ca² ⁺ may also activate calcineurin signalling, associated with fibrosis and pathological remodelling [60,61]. Together, these mechanisms may act synergistically, particularly under high steroid loads, promoting pathological cardiac remodelling as observed in the N-T group [62].

At the cellular level, these morphofunctional changes were reflected by increased cardiomyocyte width and volume in the LV, consistent with concentric hypertrophy induced by pressure overload during resistance training [20,63]. Interestingly, despite an increase in RV mass, RV cardiomyocyte morphology remained unchanged, suggesting that RV hypertrophy involves extracellular matrix remodelling and fibrosis rather than cellular hypertrophy, as previously reported [64]. This discrepancy reflects the different hemodynamic demands on each ventricle during RT, with the LV experiencing greater pressure overload and mechanical stress [20].

Echocardiographic analyses supported these findings by showing dilation of the LV systolic chamber, interventricular septum thickening, and RV diastolic enlargement exclusively in the combined ND and RT group. These structural changes were accompanied by reductions in LV ejection fraction, fractional shortening, and RV systolic function, indicating compromised cardiac performance. Such features are characteristic of early dilated cardiomyopathy and may result from pro-inflammatory and fibrotic processes triggered by high-dose ND combined with mechanical overload [56,65,66]. Together, these data demonstrate that ND and RT synergistically induce maladaptive cardiac remodelling, integrating hypertrophic cellular changes with extracellular matrix remodelling and functional impairment [61,62].

In clinical settings, an increasing number of patients have been reported to develop dilated cardiomyopathy following AAS abuse. However, the underlying pathophysiological mechanisms remain unclear [67,68]. One possible explanation for this phenomenon is that, although both ND and RT act as hypertrophic stimuli to the heart [17], excessively high doses of testosterone exert pro-inflammatory effects. This may activate genes associated with myocardial inflammation and necrosis, leading to myocarditis—a condition recognized as a potential cause of biventricular dilation and functional impairment [66]. Nevertheless, this hypothesis requires further investigation.

Contractile performance of isolated cardiomyocytes was significantly improved in N-T group, particularly in parameters such as contraction amplitude, contraction and relaxation velocities, and time to 50% relaxation. These enhancements were observed in both ventricles, even in the presence of echocardiographic signs of biventricular dilation and reduced systolic function. Such findings suggest a compensatory cellular response aimed at preserving systolic performance in the face of early myocardial dysfunction, possibly driven by extracellular matrix remodelling rather than intrinsic deficits in myocyte function [53,61].

At the molecular level, the combination of ND and TR reduced the expression of key calcium-handling proteins, notably RyR2 and PLBt in both ventricles. Although the reduction in RyR2 — a primary calcium release channel in the sarcoplasmic reticulum — could impair contractility, compensatory mechanisms preserved contractile function. One such mechanism may involve decreased PLBt expression and a lower PLB/SERCA2a ratio, which enhance SERCA2a activity and facilitate faster Ca² ⁺ reuptake into the sarcoplasmic reticulum, improving relaxation kinetics and sustaining intracellular Ca² ⁺ cycling efficiency during repeated stimulation [12,69].

These adaptations could be hypothetically explained by RT-induced phosphorylation of troponin C, which has been reported to increase Ca² ⁺ sensitivity and augment contraction, particularly under conditions of reduced RyR2 expression [70]. Although we did not directly assess troponin C phosphorylation, this mechanism may contribute to the improved contractile performance observed in our trained groups. Likewise, ND has been described in previous studies to alter the electrophysiological properties of cardiomyocytes by prolonging action potential duration and enhancing L-type Ca² ⁺ channel activity [71,72]. Such prolongation could theoretically facilitate greater Ca² ⁺ influx and reinforce excitation–contraction coupling in the short term. These effects, while potentially arrhythmogenic in vivo, may transiently enhance contractility in isolated cells, especially during the early stages of maladaptive remodelling [71].

When compared with previous studies, our findings demonstrated partial divergence. RT has been reported to enhance cardiomyocyte mechanics by increasing SERCA2a and PLBthr17 expression, decreasing total PLB, and improving Ca² ⁺ transient amplitude and kinetics [17,73–75]. In our study, RT also improved contractility but did not modify Ca² ⁺ -handling proteins, suggesting alternative mechanisms that were not explored in this paper.

Besides, the effects of AAS on cardiomyocytes are dose- and duration-dependent. Wadthaisong et al. (2019) [76] showed that 30 mg/kg/week of testosterone enhanced contraction at 4 weeks but induced dysfunction after 12 weeks. Considering total steroid load, our protocol (160 mg/kg of ND over 8 weeks vs. 120 mg/kg of testosterone over 4 weeks) produced similar early adaptations, and the combination with RT appeared synergistic.

In contrast, Seara et al. (2019) [77] reported that 10 mg/kg/week of ND for 8 weeks caused contractile impairment and altered Ca² ⁺ cycling, whereas in our sedentary ND group (double the dose) contractility was preserved despite downregulation of Ca² ⁺ -handling proteins. Moreover, Nascimento et al. (2016) [17] found that ND + RT for 4 weeks increased LV contractility without changes in SERCA2a or PLB, but with reduced NCX and PLBthr17/PLBt ratios, partially supporting our results.

Regarding redox balance, RT and ND induced oxidative stress in both cardiac ventricles, with more pronounced effects in the LV. The N-T group exhibited marked increases in MDA and PC, which indicates membrane and structural protein damage in cardiac tissue. These effects could possibly be related to previously reported ND-induced activation of NADPH oxidase via androgen receptor signalling, leading to increased reactive oxygen species generation [19,78–80].

In both ventricles, no significant changes were observed in the expression of the endogenous antioxidant enzymes SOD and CAT, indicating that classical enzymatic defenses against ROS were not effectively activated. This may reflect adaptive exhaustion of the antioxidant response due to chronic oxidative stress induced by ND, or an insufficient redox stimulus from RT to elicit a marked enzymatic response within the protocol duration [81]. Nonetheless, RT showed a trend toward increased total antioxidant capacity, with a large effect size, suggesting activation of non-enzymatic antioxidant pathways [82].

This study presents some limitations that should be addressed in future research. The lack of intracellular calcium transient measurements limits the mechanistic interpretation regarding excitation–contraction coupling. Nevertheless, the assessment of isolated cardiomyocyte contractility provided a valuable functional overview of cellular performance, allowing inferences about calcium handling at the cellular level. Moreover, to our knowledge, this is the first study to evaluate the combined effects of RT and ND on isolated cardiac cells’ contractility, highlighting the regional differences between each ventricle.

Besides, while analyses of calcium cycling proteins and redox balance markers provided valuable mechanistic insights, they do not fully clarify the underlying pathways. Future studies are encouraged to explore post-transcriptional and post-translational mechanisms, including the potential involvement of epigenetic regulators, such as microRNAs. Profiling miRNA expression could help identify a specific molecular fingerprint of AAS-induced cardiac remodelling, shedding light on the lasting molecular alterations promoted by these compounds [83].

## Conclusion

Despite being a preclinical investigation with inherent limitations for direct extrapolation to humans, this study provides important insights into the molecular and pathophysiological effects of AAS combined with resistance training on cardiac function. The results indicate that AAS use, even alongside physical training, can induce pathological cardiac remodelling, oxidative stress, and impaired calcium handling, potentially explaining cardiovascular complications observed in athletes and active individuals abusing these substances. These findings emphasize the need for prevention efforts and can inform future clinical research and interventions aimed at mitigating cardiovascular risks related to AAS misuse.

In summary, the combination of RT and ND induced ventricular hypertrophy and structural remodelling that impaired overall cardiac function in Wistar rats. Despite improvements in cardiomyocyte contractile parameters —novel findings not previously reported — these adaptations occurred alongside reduced expression of calcium-regulatory proteins and increased oxidative stress markers, indicating a maladaptive cardiac response. These results highlight a previously unrecognized dissociation between functional and molecular remodelling, with potential implications for athletes and clinical populations exposed to anabolic steroids and high-intensity resistance training.
